# Effectiveness of plasma lyso-Gb3 as a biomarker for selecting high-risk patients with Fabry disease from multispecialty clinics for genetic analysis

**DOI:** 10.1038/gim.2018.31

**Published:** 2018-03-15

**Authors:** Hiroki Maruyama, Kaori Miyata, Mariko Mikame, Atsumi Taguchi, Chu Guili, Masaru Shimura, Kei Murayama, Takeshi Inoue, Saori Yamamoto, Koichiro Sugimura, Koichi Tamita, Toshihiro Kawasaki, Jun Kajihara, Akifumi Onishi, Hitoshi Sugiyama, Teiko Sakai, Ichijiro Murata, Takamasa Oda, Shigeru Toyoda, Kenichiro Hanawa, Takeo Fujimura, Shigehisa Ura, Mimiko Matsumura, Hideki Takano, Satoshi Yamashita, Gaku Matsukura, Ryushi Tazawa, Tsuyoshi Shiga, Mio Ebato, Hiroshi Satoh, Satoshi Ishii

**Affiliations:** 1 Department of Clinical Nephroscience, Niigata University Graduate School of Medical and Dental Sciences, Niigata, Japan; 2 Sanofi K.K., Sanofi Genzyme Medical Operations, Rare Disease Medical, Medical Science Liaison, Tokyo, Japan; 3 Department of Metabolism, Chiba Children’s Hospital, Chiba, Japan; 4 Department of Pediatrics, Dokkyo Medical University Koshigaya Hospital, Koshigaya, Japan; 5 Department of Cardiovascular Medicine, Tohoku University Graduate School of Medicine, Sendai, Japan; 6 Nishinomiya Watanabe Cardiovascular Center, Nishinomiya, Japan; 7 Department of Cardiology, Fujinomiya City General Hospital, Fujinomiya, Japan; 8 Department of Human Resource Development of Dialysis Therapy for Kidney Disease, Okayama University Graduate School of Medicine, Dentistry and Pharmaceutical Science, Okayama, Japan; 9 Sukoyaka Clinic, Gifu, Japan; 10 Department of Chronic Kidney Disease, Gifu University Graduate School of Medicine, Gifu, Japan; 11 Yamaguchi Prefectural Grand Medical Center, Hofu, Japan; 12 Department of Cardiovascular Medicine, Dokkyo Medical University, Mibu, Japan; 13 Department of Cardiology, Internal Medicine, Iwaki Kyoritsu General Hospital, Iwaki, Japan; 14 Department of Nephrology, Kashiwazaki General Hospital and Medical Center, Kashiwazaki, Japan; 15 Division of Neurology, Japanese Red Cross Asahikawa Hospital, Asahikawa, Japan; 16 Department of Nephrology, Tokyo Teishin Hospital, Kashiwazaki, Japan; 17 Department of Cardiology, Japanese Red Cross Hamamatsu Hospital, Hamamatsu, Japan; 18 Division of Medical Genetics, Bioscience Medical Research Center, Niigata University Medical and Dental Hospital, Niigata, Japan; 19 Department of Cardiology, Tokyo Women’s Medical University, Tokyo, Japan; 20 Division of Cardiology, Showa University Fujigaoka Hospital, Yokohama, Japan; 21 Division of Cardiology, Internal Medicine III, Hamamatsu University School of Medicine, Hamamatsu, Japan; 22 GlycoPharma Corporation, Oita, Japan

**Keywords:** Fabry disease, gene analysis, genetic variants of uncertain significance, lyso-Gb3, screening

## Abstract

**Purpose:**

Plasma globotriaosylsphingosine (lyso-Gb3) is a promising secondary screening biomarker for Fabry disease. Here, we examined its applicability as a primary screening biomarker for classic and late-onset Fabry disease in males and females.

**Methods:**

Between 1 July 2014 and 31 December 2015, we screened 2,360 patients (1,324 males) referred from 169 Japanese specialty clinics (cardiology, nephrology, neurology, and pediatrics), based on clinical symptoms suggestive of Fabry disease. We used the plasma lyso-Gb3 concentration, α-galactosidase A (α-Gal A) activity, and analysis of the *α-Gal A* gene (*GLA*) for primary and secondary screens, respectively.

**Results:**

Of 8 males with elevated lyso-Gb3 levels (≥2.0 ng ml^–1^) and low α-Gal A activity (≤4.0 nmol h^–1^ ml^–1^), 7 presented a *GLA* mutation (2 classic and 5 late-onset). Of 15 females with elevated lyso-Gb3, 7 displayed low α-Gal A activity (5 with *GLA* mutations; 4 classic and 1 late-onset) and 8 exhibited normal α-Gal A activity (1 with a classic *GLA* mutation and 3 with genetic variants of uncertain significance).

**Conclusion:**

Plasma lyso-Gb3 is a potential primary screening biomarker for classic and late-onset Fabry disease probands.

## Introduction

Fabry disease (FD) is an X-linked lysosomal storage disorder that results from a deficiency in the activity of α-galactosidase A (α-Gal A).^1^ The α-Gal A deficiency causes systemic lysosomal accumulation of glycolipids, predominantly globotriaosylceramide (Gb3), in the vascular endothelium and other tissues. Morbidity and mortality from FD—caused by renal failure, cardiac disease, and early-onset stroke—increase with age. Cardiac deaths account for most FD-related deaths in females and males.^2^ FD can be classified as classic or late-onset.^3^ Late-onset FD lacks classic early manifestations, such as acroparesthesia, clustered angiokeratoma, cornea verticillata, and hypo-anhidrosis, and exhibits exclusively renal, cardiac, and cerebral impairments. Thus, recognizing late-onset FD is difficult, and undiagnosed patients with late-onset FD may outnumber those with classic FD.^4^

FD targets multiple organs, and its effective treatment requires the efforts of doctors from several clinical departments. Nephrologists, cardiologists, and neurologists are important for managing both classic and late-onset FD. Previously, nephrologists and cardiologists have accidentally identified late-onset FD probands when pathological findings from kidney and cardiac biopsies were consistent with FD, and the *α-Gal A* gene (*GLA*) mutation analysis was then used to confirm the diagnosis of FD.^3^ However, such invasive biopsies are rare, and many cases of FD may go undiagnosed. Thus, it is important to find reliable and noninvasive biomarkers for FD screening.

There are many genetic variants of uncertain significance (GVUS) in *GLA*.^5^ Therefore, *GLA* analysis is not always the gold standard for diagnosis in all cases. *GLA* mutations can be divided into two classes: class 1 mutations, with a high probability of causing disease, and class 2 mutations representing nonpathogenic polymorphisms.^6^ *GLA* analysis can definitively identify FD by class 1 mutations, or non-FD by class 2 mutations.

*GLA* analysis is generally considered only when the patient presents with abnormal α-Gal A activity and clinical symptoms that suggest FD. Moreover, α-Gal A activity often remains within the normal range in female patients,^7^ making FD diagnosis more difficult.^3^

The prevalence rates of FD in Japan, as determined by α-Gal A activity screening, were 0.2% for patients with classic FD, 1.0% for late-onset FD in male patients undergoing dialysis,^8,9^ and 3% for late-onset FD in patients with male left ventricular hypertrophy (LVH).^10^ In a recent systematic review of high-risk populations for FD, the prevalence of a definitive diagnosis of FD was 0.12% for classic FD.^5^

The deacylated form of Gb3, globotriaosylsphingosine (lyso-Gb3), was recently identified in plasma as a strong biomarker of classic FD, in studies of recognized patients with FD who had already been diagnosed.^11,12,13^ However, the usefulness of lyso-Gb3 for proband screening has not been studied. Previously, we screened for FD in male patients undergoing dialysis, using plasma α-Gal A activity as the primary biomarker and lyso-Gb3 concentration, as measured by high-performance liquid chromatography, as a secondary biomarker. This was the first study to investigate whether lyso-Gb3 could be used as a secondary biomarker to identify male probands in an FD-suspected population. We found that the plasma lyso-Gb3 screening was effective for selecting candidates for genetic counseling and testing, revealing unrecognized FD cases, and reducing the number of unnecessary gene analyses.^14^

In the present study, we assessed the value of lyso-Gb3 as an FD biomarker for screening male and female patients suspected of having FD based on clinical symptoms. We screened for the concentration of lyso-Gb3, as measured by ultra-performance liquid chromatography/tandem mass spectrometry, and for the plasma α-Gal A activity in all patients. Although previous studies evaluated patients already diagnosed with FD,^12^ this is the first study to investigate whether lyso-Gb3 could be used in screening assays as the primary biomarker to identify male and female probands in patients with suspected FD. Plasma lyso-Gb3 could be used in the selection of candidates who may benefit from gene analysis, to improve the outcomes of diagnosis in multispecialty clinics.

## Materials and methods

### Study design

This was a prospective multicenter study.

*Lyso-Gb3 and α-Gal A screening*

This study had four steps: (i) patients were screened for FD by measuring the concentration of plasma lyso-Gb3 (≥2.0 ng ml^–1^: positive) and plasma α-Gal A activity (activity ≤4.0 nmol h^–1^ ml^–1^: low); (ii) patients with elevated lyso-Gb3 or low α-Gal A activity were informed that they may have FD; (iii) with the consent of the patient, the FD diagnosis was confirmed by genetic testing using *GLA*; and (iv) a familial diagnosis was confirmed by screening other family members based on the lyso-Gb3 concentration, α-Gal A activity, and *GLA* analysis when indicated.

*Patient enrollment*

Japanese patients of unknown FD status who had been referred by specialty clinics were screened for FD between 1 July 2014 and 31 December 2015. This study is ongoing; the interim results, up to 2015, are presented here. Patients (aged 2–99 years) suspected of having FD based on clinical evaluation were recruited at 169 clinics in Japan. Clinical evaluation included: nephrological evaluation with biochemical examination, urinalysis, imaging, and kidney biopsy (for chronic kidney disease,^15^ unexplained proteinuria, or pathological findings consistent with FD); cardiac evaluation including an electrocardiogram, echocardiogram, and cardiac magnetic resonance imaging (unexplained LVH or unexplained cardiac failure); neurological evaluation with magnetic resonance imaging (early-onset stroke or transient ischemic attack); and pediatric evaluation in children with early classic manifestations, such as acroparesthesia, clustered angiokeratoma, cornea verticillata, and hypo-anhidrosis. Acroparesthesia is defined as pain in the hands and/or feet, with the onset of pain in childhood or adolescence, and/or a course characterized by exacerbations that are provoked by fever, exercise, or heat, as well as decreased cold sensation.^16^ Clustered angiokeratoma should be present in the bathing trunk, periumbilical, and/or perioral regions.^16^ Cornea verticillata should be evaluated using a slit lamp, in the absence of amphiphilic drug use.^16^ Hypo-anhidrosis is defined as low or no sweating even in an environment (high temperature or humidity) that encourages sweating. Patients with known FD and their relatives were excluded.

*Informed consent*

This study was conducted in accordance with the Declaration of Helsinki. The study protocol was approved by the ethics committee of the Niigata University School of Medicine (permit number: 1802, 2367, H25–661, H27–805) and collaborating clinics. Patients were informed about the study specifics, and those who agreed to participate provided written consent. The results were reported to the patient by the attending doctor. Informed consent was also obtained from patients before *GLA* analysis.

### Sample collection

Blood specimens for the primary screening were collected in Venoject II collection tubes (Terumo; Tokyo, Japan). Plasma samples were obtained by centrifugal separation.

### Measurement of plasma lyso-Gb3

Plasma lyso-Gb3 levels were measured by ultra-performance liquid chromatography/tandem mass spectrometry at GlycoPharma (Oita, Japan), with a detection threshold of 0.1 ng ml^–1^ (Supplementary Methods online). In a previous study,^17^ the cutoff for lyso-Gb3 levels was 0.9 ng ml^–1^ (ninety-fifth percentile of healthy individuals), with the highest normal value at 2.0 ng ml^–1^. To avoid false-positive results, we set the screening cutoff for lyso-Gb3 levels at 2.0 ng ml^–1^. We evaluated the suitability of this high cutoff value (2.0 ng ml^–1^) for both genders; the highest concentration of lyso-Gb3 among males with normal α-Gal A activity in this study was 1.8 ng ml^–1^.

### Measurement of plasma α-Gal A activity

Plasma α-Gal A activity was measured at GlycoPharma using the artificial substrate 4-methylumbelliferyl-α-d-galactoside, with a detection threshold of 0.1 nmol h^–1^ ml^–1^, as described previously.^14^ To avoid false-negative results, the screening cutoff for the α-Gal A activity level—the percentage of the control mean or cohort mean—was recently set at high, ≥50%.^14^ We did not set up reference individuals for plasma α-Gal A activity. Therefore, reference values for plasma α-Gal A activity were not obtained. The cutoff for a low result for plasma α-Gal A activity was set at 4.0 nmol h^–1^ ml^–1^—54% of the mean cohort value in our previous study.^14^

### Genetic counseling

Patients positive for lyso-Gb3 or showing low α-Gal A values were considered candidates for genetic counseling. The results of the α-Gal A activity screening were explained to the participants or family members, as appropriate.

### Sample collection for gene analysis

To obtain DNA and RNA samples, blood specimens were collected in Venoject II collection tubes and PAXgene Blood RNA Tubes (PreAnalytiX; Hombrechtikon, Switzerland), respectively.

### Gene analysis

Gene analysis was performed at the Department of Clinical Nephroscience, Niigata University Graduate School of Medical and Dental Sciences (Supplementary Methods and Supplementary Tables S1–S3 online).

### Statistical analysis and graph preparation

The data were examined using the Shapiro–Wilk test to determine whether they showed a normal distribution. The majority of the continuous variables were non-normally distributed and therefore the medians and interquartile ranges (IQRs) are presented. Categorical data were evaluated by Fisher’s exact test and Pearson’s chi-squared test. All reported significant values were two-tailed. Data were compared between two unpaired groups by performing the Wilcoxon rank-sum test. JMP 12 software (SAS Institute; Cary, NC) was used for statistical analyses, and the results were considered significant at *P* < 0.05. Data were plotted using SigmaPlot 12.5 (Systat Software; San Jose, CA).

## Results

### Study population

We enrolled 2,376 patients suspected of having FD based on clinical symptoms. We excluded 16 patients, including 2 patients who were known to have FD and 14 patients with relatives who were known to have FD. We obtained validated data for 2,360 patients (1,324 males and 1,036 females) from 169 clinics (Table 1). The median patient age when blood samples were obtained was 64 years for males (IQR: 50–72) and 67 years for females (IQR: 54–77).

**Table 1:** Patients grouped according to the medical specialty of the referring clinic

	**Cardiology**	**Nephrology**	**Neurology**	**Pediatrics**	**Total**
**Males**	**(** *n* ** = 511)**	**(** ***n*** ** = 508)**	**(** ***n*** ** = 286)**	**(** ***n*** ** = 19)**	**(** ***n*** ** = 1,324)**
Median age (years)	65	67	54	11	64
IQR (years)	52–72	57–74	45–67	9–13	50–72
Clinics (*n*)	48	22	27	8	105
**Females**	**(** ***n*** ** = 224)**	**(** ***n*** ** = 550)**	**(** ***n*** ** = 227)**	**(** ***n*** ** = 35)**	**(** ***n*** ** = 1,036)**
Median age (years)	66	71	55	12	67
IQR (years)	53–74	62–79	45–76	9–14	54–77
Clinics (*n*)	53	33	40	21	147

IQR, interquartile range.

### Plasma lyso-Gb3 levels

**Figure 1:**
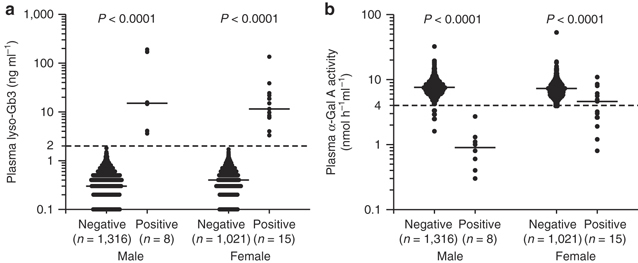
**Plasma globotriaosylsphingosine (lyso-Gb3) levels and α-galactosidase A (α-Gal A) activity in male and female participants.** Patients were classified according to plasma lyso-Gb3 levels: <2.0 ng ml^–1^ (negative) or ≥2.0 ng ml^–1^ (positive). (**a**) Dotted line: 2.0 ng ml^–1^. Short horizontal lines indicate the median plasma lyso-Gb3 value in each group. When plasma lyso-Gb3 values were less than the detection threshold (0.1 ng ml^–1^), a value of 0 ng ml^–1^ was used to represent the lyso-Gb3 levels in the statistical analysis. Zero cannot be plotted on a logarithmic graph. Undetectable plasma lyso-Gb3 levels of 166 males and 134 females in each negative group were not plotted on the graph. (**b**) Dotted line: 4.0 nmol h^–1^ ml^–1^. Short horizontal lines indicate the median plasma α-Gal A activity in each group. Plasma α-Gal A activities were detected in all patients.

Based on plasma lyso-Gb3 levels, patients were classified as positive (≥2.0 ng ml^–1^) or negative (<2.0 ng ml^–1^). The median lyso-Gb3 levels were 15.1 ng ml^–1^ in the 8 males in the positive group (IQR: 4.0–171.4) and 0.3 ng ml^–1^ in the 1,316 males in the negative group (IQR: 0.2–0.5); this difference was significant (*P* < 0.0001; Figure 1a). The median lyso-Gb3 levels were 11.5 ng ml^–1^ for the 15 females in the positive group (IQR: 7.8–21.8) and 0.4 ng ml^–1^ for the 1,021 females in the negative group (IQR: 0.2–0.6); this difference was also significant (*P* < 0.0001; Figure 1a).

### Plasma α-Gal A activity

The median plasma α-Gal A activity in the 8 males in the positive group was 0.9 nmol h^–1^ ml^–1^ (IQR: 0.5–1.3), which was significantly lower than the median of 7.6 nmol h^–1^ ml^–1^ (IQR: 6.6–8.9) in the 1,316 males in the negative group (*P* < 0.0001; Figure 1b). The α-Gal A activity was low in all 8 males in the positive group and 11 males in the negative group. The median α-Gal A activity in the 15 females in the positive group was 4.6 nmol h^–1^ ml^–1^ (IQR: 2.6–6.1), which was significantly lower (*P* < 0.0001; Figure 1b) than in the 1,021 females in the negative group (7.2 nmol h^–1^ ml^–1^; IQR: 6.1–8.5). Eight females in the positive group had normal α-Gal A activity, and six females in the negative group had low α-Gal A activity. Thus, the relationship between plasma lyso-Gb3 levels and α-Gal A activity differed between males and females.

### Classification of FD probands with class 1 mutations

Ordinary gene analysis revealed that 13 of the FD probands (7 males and 6 females) had class 1 mutations. These probands were classified as classic or late-onset FD based on the presence or absence of early classic manifestations and the type of *GLA* mutation. Among the cases of FD identified in this study, we found ten previously reported *GLA* mutations (p.L68F,^18^ p.R301Q,^19^ p.Q312R,^20^ p.R112H,^21^ p.K391E,^22^ p.R220P,^23^ p.D231N,^24^ p.L415P,^25^ p.N263S,^26^ and p.L403S).^20^ Two other mutations were identified—i.e., p.G85V and the frameshift mutation c.559_560delAT—and both probands were classified as the classic type. Thus, lyso-Gb3 screening identified both classic and late-onset FD probands in males and females (Table 2).

**Table 2:** Characterization of patients with Fabry disease

	**Patient no.**	**Lyso-Gb3 level (ng ml** ^**–1**^)	**α-Gal A activity (nmol h** ^**–1**^ ** ml** ^**–1**^)	***GLA*** **mutation**	**Age (years)**	**Classic manifestation**	**Heart**	**Kidney** ^a^	**Central nervous system**
**Males; classic type**									
Pediatrics	1	190.2	0.4	p.L68F	13	Acroparesthesia; hypohidrosis			
	2	172.2	0.3	p.G85V	9	Acroparesthesia; hypohidrosis			
**Males; late-onset type**									
Cardiology	3	15.6	1.1	p.R301Q	55		LVH		
	4	14.5	1.0	p.Q312R	77		LVH		
	5	3.6	2.7	p.R112H	61		LVH	G5DA1	
Nephrology	6	4.1	0.6	p.R112H	42			G2A3	
	7	4.0	1.3	p.K391E	75			G5DA2	Stroke
**Females; classic type**									
Cardiology	8	24.2	1.6	p.R220P	65	Angiokeratoma; cornea verticillata	LVH	G3bA1	Stroke
	9	21.8	3.2	c.559_560 delAT	65	Acroparesthesia; gastrointestinal symptoms	LVH		Stroke
	10	4.0	8.0	p.D231N	63		Arrhythmia; heart failure		
Nephrology	11	15.0	2.6	p.L415P	32	Acroparesthesia	LVH	G1A3	
Neurology	12	135.0	0.8	p.N263S (XO)	27	Acroparesthesia; hypohidrosis; angiokeratoma	Arrhythmia	G1A3	
**Females; late-onset type**									
Nephrology	13	3.3	2.6	p.L403S	53		LVH	G5A3	

LVH, left ventricular hypertrophy; XO, gonadal dysgenesis.

^a^Glomerular filtration rate category [ml min^−1^ (1.73m^2^)^−1^]: G1, ≥90; G2, 60–89; G3a, 45–59; G3b, 30–44; G4, 15–29; G5, <15; D, dialysis^15^. Proteinuria category (urinary protein-to-creatinine ratio; g/gCr): A1, <0.15; A2, 0.15–0.49; A3, ≥0.50.

All the male FD probands were in the positive group and had low α-Gal A activity. Of the two male probands with classic FD, both were pediatric patients (Table 2). Of the five probands with late-onset FD, three were referred from cardiology and two from nephrology (Table 2). All the female FD probands were in the positive group, and all but one had low α-Gal A activity. Of the five female probands with classic FD, three were referred from cardiology, one from nephrology, and one from neurology (Table 2). One female proband with Turner’s syndrome (gonadal dysgenesis) and a classic-type mutation presented high lyso-Gb3 levels, similar to those found in males with classic FD (Table 2). There was one female late-onset FD proband, and she was referred from nephrology (Table 2).

### Relationships between the classification of FD and age or plasma lyso-Gb3 levels in the probands

**Figure 2:**
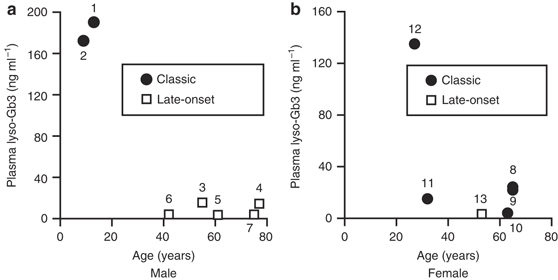
**Relationships between Fabry disease classification and age or plasma globotriaosylsphingosine (lyso-Gb3) level in male and female probands.** Class 1 mutations were confirmed in all probands. (**a**) Male classic- (*n* = 2) and late-onset-type (*n* = 5) probands. (**b**) Female classic- (*n* = 5) and late-onset type (*n* = 1) probands. Numbers beside data points correspond to the patient numbers in Table 2.

All male probands with classic FD were children, and those with late-onset FD were adults (Figure 2a). The lyso-Gb3 levels were markedly higher in the male probands with classic FD than those with late-onset FD (Figure 2a). Female probands with classic FD had a wide age range (Figure 2b), and no differences were observed in lyso-Gb3 levels between females with classic FD and those with late-onset FD (Figure 2b).

### Frequency of FD diagnosis by clinical department following positive lyso-Gb3 screens

In all clinical departments except pediatrics, the frequency of identification among patients who screened positive for lyso-Gb3 was higher in females than in males (Table 3). Of the referring departments that treated adult patients, cardiology had the highest frequency of diagnosis among females who screened positive for lyso-Gb3 (Table 3).

**Table 3:** Frequencies of patients who screened positive for lyso-Gb3 and class 1 *GLA* mutations, grouped according to the specialty of the referring clinic

	**Cardiology**	**Nephrology**	**Neurology**	**Pediatrics**	**Total**
**Males**					
Lyso-Gb3-positive patients (%)	3/511 (0.6)	3/508 (0.6)	0/286 (0)	2/19 (10.5)	8/1,324 (0.6)
*GLA* mutation (%)	3/511 (0.6)	2/508 (0.4)	0/286 (0)	2/19 (10.5)	7/1,324 (0.5)
Classic type	0	0	0	2	2
Late-onset type	3	2	0	0	5
**Females**					
Lyso-Gb3-positive patients (%)	8/224 (3.6)	5/550 (0.9)	2/227 (0.9)	0/35 (0)	15/1,036 (1.4)
*GLA* mutation (%)	3/224 (1.3)	2/550 (0.4)	1/227 (0.4)	0/35 (0)	6/1,036 (0.6)
Classic type	3	1	1	0	5
Late-onset type	0	1	0	0	1

### Additional *GLA* analysis for females who screened positive for lyso-Gb3, but for whom ordinary gene analysis failed to detect class 1 mutations

Unexpectedly, ordinary gene analyses were unable to find putative mutations in three females who exhibited normal α-Gal A activity and screened positive for lyso-Gb3 (Supplementary Table S4 online). Multiplex ligation-dependent probe amplification analyses did not reveal any copy-number differences. Intronic analyses revealed many GVUS (Supplementary Table S5). However, it was difficult to distinguish the major *GLA* variants associated with FD. Electron microscopy revealed lamellar bodies in kidney or endomyocardial biopsies (Supplementary Table S4). The endomyocardium of patients 14 and 15 could be observed with high magnification. Their lamellar bodies showed the characteristic storage of Gb3, which was defined by concentric multilamellated myelin bodies in a zebra-like pattern, with a periodicity of approximately 5 nm.^16^ None of the patients showed any symptoms that would indicate Niemann–Pick disease, nor were they treated with amiodarone, chloroquine, or tamoxifen, all of which may lead to drug-induced phospholipidosis. Together, these data indicate a probable diagnosis of FD.

### Ordinary gene analyses and subsequent intronic analyses for patients who screened negative for lyso-Gb3 and displayed low α-Gal A activity

To determine whether the lyso-Gb3 screening identified all FD cases in the study population, we conducted gene analyses for patients who had screened negative for lyso-Gb3 and displayed low α-Gal A activity. In total, 17 of our patients (11 males and 6 females) met these criteria. Of the 11 males, 7 declined gene analysis. Of the four males who underwent ordinary gene analysis, three presented the p.E66Q class 2 mutation—a nonpathogenic, functional polymorphism that was found at an unexpectedly high frequency in the *GLA* analysis of Japanese patients selected through the abnormal α-Gal A activity in the FD screening.^14,27^ One male with normal plasma lyso-Gb3 levels exhibited a gene promoter variant (c.−10C>T) in the 5'-untranslated region of exon 1 that was associated with decreased α-Gal A expression;^28^ this is a nonpathogenic class 2 mutation (Supplementary Table S6 online).^28^ Of the six females, four declined gene analysis and the two who underwent ordinary gene analysis presented the c.−10C>T mutation (Supplementary Table S6).

A subsequent intronic mutation analysis in three patients with c.−10C>T revealed many GVUS (Supplementary Table S7).

### Flowcharts of the results of lyso-Gb3 screening and gene analysis

The results of this study are summarized in flowcharts (Supplementary Figures S1 and S2 online). We found classic and late-onset FD probands in both males and females who screened positive for elevated lyso-Gb3 levels.

## Discussion

In this study, we assessed the applicability of lyso-Gb3 as an FD biomarker for screening male and female patients suspected of FD based on clinical symptoms. This method enabled us to uncover both classic and late-onset FD probands. This is the first assessment of the potential use of lyso-Gb3 as a primary screening biomarker. Our findings show that plasma lyso-Gb3 might represent a promising primary screening biomarker for identifying FD probands.

Lyso-Gb3 screening was effective for identifying male FD probands. Males who screened negative for lyso-Gb3 and displayed low α-Gal A activity presented the p.E66Q or c.−10C>T mutations. This result indicates that our current screening method—using lyso-Gb3 determined by ultra-performance liquid chromatography/tandem mass spectrometry—might exclude patients with the p.E66Q mutation. Thus, FD could be excluded in male patients with normal lyso-Gb3 levels.^29^

Among male patients examined in this study, elevated lyso-Gb3 levels were suggestive of a diagnosis of FD. Males with classic FD exhibited higher lyso-Gb3 levels than those with late-onset FD. Therefore, it should be possible to establish a reliable lyso-Gb3 cutoff value for the classification of naive hemizygous patients with FD. A lyso-Gb3 value above 45 nmol l^–1^ predicts a diagnosis of classic FD in males.^29^ Similarly, a cutoff value around 50 nmol l^–1^ for the classic FD classification was reported, with lower levels found in late-onset FD except for one patient who presented a level of 74 nmol l^–1^.^12^ The integration of additional screening data will help to establish the lower levels found in classic FD and the upper levels found in late-onset FD, to determine the boundaries defining these forms of FD.

Lyso-Gb3 screening also identified female FD probands, and has the potential to identify many unrecognized female probands. Our pedigree analyses showed that lyso-Gb3 levels in asymptomatic females of families carrying class 1 mutations did not exceed 2.0 ng ml^–1^ (data not shown; details to be reported separately). Lyso-Gb3 levels of female patients with FD overlap with controls.^29^ Therefore, setting a lower threshold for females (below 2 ng ml^–1^) might prove tricky. A robust analysis of lyso-Gb3 in both FD and other lysosomal storage disorders is needed to be absolutely certain regarding the specificity of setting a lower threshold for female patients. In this study, we found that setting a cutoff value to classify even naive heterozygous patients with FD may prove difficult, and lyso-Gb3 values in familial hemizygotes might help in the classification of female probands.

Determining a top-priority target is fundamental for developing a successful screening strategy for high-risk populations. In this study, we found a high prevalence of FD in females referred from cardiology (1.3%). The incidence of LVH was particularly high in older female patients with FD, and the cumulative incidence of LVH in females over 68 years of age was 100%.^30^ For females, plasma lyso-Gb3 represents an independent risk factor for developing LVH.^13^ Thus, screening of females with LVH is essential. Most patients identified in previous screens for FD in patients with early stroke^31^ exhibited class 2 mutations rather than FD. The actual frequency of patients identified was lower than the reported frequency.^31^ Similarly, the identification frequencies in the recent report^32^ and the present study were low.

Screening for FD in high-risk populations yields many individuals with GVUS.^5^ Intronic *GLA* variants often remain unidentified because these regions are not evaluated by ordinary gene analyses, except for exon–intron boundaries. Surprisingly, intronic analyses in the current study identified patients with multiple GVUS. Combinations of c.−10C> and nonpathogenic intronic variants might be associated with FD.^33–35^ Although *GLA* analysis is our most reliable method for diagnosing FD, diagnostic clarity may remain elusive when GVUS are present. Elucidating the roles of various intronic GVUS will improve the effectiveness of lyso-Gb3 screening.

Some issues arise in the diagnosis of FD. Thus, the α-Gal A activity assay is not reliable for females. Moreover, since late-onset FD lack the early classic manifestations, patients with late-onset FD may be less likely to be diagnosed than those with classic FD and may be dormant. A previous study has already shown that lyso-Gb3 levels are related to FD phenotype.^29^ When patients are phenotyped according to well-defined criteria, overlap only occurs between the controls and female patients with non-classical FD.^29^ Hence, using lyso-Gb3 to confirm a diagnosis of FD may represent a good approach in females and patients with late-onset FD. Another issue is that subjects with an uncertain diagnosis of FD who present non-specific FD symptoms and GVUS are increasingly identified through screening.^5^ Both the confirmation and exclusion of FD are important for an adequate diagnosis. A diagnostic algorithm for these subjects was recently proposed.^5,16^ The gold standard for a diagnosis of FD in these subjects involves evidence of a specific storage pattern in the affected organ, as assessed by electron microscopic observation with high magnification.^5,16^

In this study, we found that some female patients with high lyso-Gb3 levels and normal α-Gal A screening results presented intronic GVUS; this association was revealed exclusively through lyso-Gb3 screening. Although no clear diagnostic criteria exist for GVUS, high lyso-Gb3 levels support a likelihood of FD and might encourage subsequent histological examinations to reach a definitive diagnosis.

Female patients with classic^36^ and late-onset FD,^37^ and male patients with late-onset FD^38^ passed fitness checks for kidney donation, but were later diagnosed with FD based on graft biopsy findings. Obtaining a kidney biopsy from a donor candidate before transplantation is difficult. Screening for lyso-Gb3 along with a genetic analysis may offer a safeguard against transplanting an inadequate kidney from an asymptomatic female with classic FD or a donor with late-onset FD.

Recently, genetic screening for FD in 2,034 probands referred from multispecialty clinics in a 10-year prospective study showed a significant increase in diagnostic yield (1.3%).^39^ Namely, more than 98% of patients did not have FD and underwent gene analysis. Additionally, data on lyso-Gb3 levels were not included. We believe that reducing the number of patients without FD that undergo gene analysis is important for patients’ peace of mind. This study revealed that the frequencies of positive lyso-Gb3 screening were 0.6% in males and 1.4% in females. If all patients had undergone *GLA* analysis, the prevalence of FD may have exceeded 0.55%, approaching the rate obtained from genetic screening. Thus, plasma lyso-Gb3 analysis may allow the selective identification of patients who are at high risk for FD.

This study has several limitations. First, not all patients were genotyped; therefore, some cases of FD may have been missed. Second, normal lyso-Gb3 concentrations do not exclude FD in females, and some cases—particularly late-onset FD—may have been missed.^29^ Third, plasma lyso-Gb3 concentrations are elevated in boys with classic FD from infancy onwards, but increase gradually in girls as they get older.^11^ Therefore, some cases of FD in girls may have been missed. Nonetheless, lyso-Gb3 screening successfully identified several FD probands. Fourth, we did not establish a wide range for the age (2–99 years)-matched control population without class 1 mutations. Therefore, we could not compare the pathological lyso-Gb3 levels with the control population. Plasma lyso-Gb3 is a promising primary screening biomarker that effectively identified unrecognized FD probands referred from multispecialty clinics.

## Electronic supplementary material

Supplementary File

## Electronic supplementary material

Supplementary material is linked to the online version of the paper at 10.1038/gim.2018.31
