# Correction: Effectiveness of plasma lyso-Gb3 as a biomarker for selecting high-risk patients with Fabry disease from multispecialty clinics for genetic analysis

**DOI:** 10.1038/s41436-018-0258-3

**Published:** 2018-09-12

**Authors:** Hiroki Maruyama, Kaori Miyata, Mariko Mikame, Atsumi Taguchi, Chu Guili, Masaru Shimura, Kei Murayama, Takeshi Inoue, Saori Yamamoto, Koichiro Sugimura, Koichi Tamita, Toshihiro Kawasaki, Jun Kajihara, Akifumi Onishi, Hitoshi Sugiyama, Teiko Sakai, Ichijiro Murata, Takamasa Oda, Shigeru Toyoda, Kenichiro Hanawa, Takeo Fujimura, Shigehisa Ura, Mimiko Matsumura, Hideki Takano, Satoshi Yamashita, Gaku Matsukura, Ryushi Tazawa, Tsuyoshi Shiga, Mio Ebato, Hiroshi Satoh, Satoshi Ishii

**Affiliations:** 1 Department of Clinical Nephroscience, Niigata University Graduate School of Medical and Dental Sciences, Niigata, Japan; 2 Sanofi K.K., Sanofi Genzyme Medical Operations, Rare Disease Medical, Medical Science Liaison, Tokyo, Japan; 3 Department of Metabolism, Chiba Children’s Hospital, Chiba, Japan; 4 Department of Pediatrics, Dokkyo Medical University Koshigaya Hospital, Koshigaya, Japan; 5 Department of Cardiovascular Medicine, Tohoku University Graduate School of Medicine, Sendai, Japan; 6 Nishinomiya Watanabe Cardiovascular Center, Nishinomiya, Japan; 7 Department of Cardiology, Fujinomiya City General Hospital, Fujinomiya, Japan; 8 Department of Human Resource Development of Dialysis Therapy for Kidney Disease, Okayama University Graduate School of Medicine, Dentistry and Pharmaceutical Science, Okayama, Japan; 9 Sukoyaka Clinic, Gifu, Japan; 10 Department of Chronic Kidney Disease, Gifu University Graduate School of Medicine, Gifu, Japan; 11 Yamaguchi Prefectural Grand Medical Center, Hofu, Japan; 12 Department of Cardiovascular Medicine, Dokkyo Medical University, Mibu, Japan; 13 Department of Cardiology, Internal Medicine, Iwaki Kyoritsu General Hospital, Iwaki, Japan; 14 Department of Nephrology, Kashiwazaki General Hospital and Medical Center, Kashiwazaki, Japan; 15 Division of Neurology, Japanese Red Cross Asahikawa Hospital, Asahikawa, Japan; 16 Department of Nephrology, Tokyo Teishin Hospital, Tokyo, Japan; 17 Department of Cardiology, Japanese Red Cross Hamamatsu Hospital, Hamamatsu, Japan; 18 Division of Medical Genetics, Bioscience Medical Research Center, Niigata University Medical and Dental Hospital, Niigata, Japan; 19 Department of Cardiology, Tokyo Women’s Medical University, Tokyo, Japan; 20 Division of Cardiology, Showa University Fujigaoka Hospital, Yokohama, Japan; 21 Division of Cardiology, Internal Medicine III, Hamamatsu University School of Medicine, Hamamatsu, Japan; 22 Glyco Pharma Corporation, Oita, Japan

Correction to: *Genetics in Medicine*; 10.1038/gim.2018.31; published online 15 March 2018

The PDF and HTML versions of the article have been updated to include the Creative Commons Attribution 4.0 International License information.

