# Supplementary material for: Effectiveness of plasma lyso-Gb3 as a biomarker for selecting high-risk patients with Fabry disease from multispecialty clinics for genetic analysis
Source: Genet Med. 2018 Mar 15;21(1):44–52. doi: 10.1038/gim.2018.31 (PMC6363642; doi:10.1038/gim.2018.31)
Supplement: Supplementary file 1 — Supplementary File [file 41436_2018_239_MOESM1_ESM.pdf]

Effectiveness of plasma lyso-Gb3 as a biomarker for selecting high-risk patients with Fabry disease from multispecialty clinics for genetic analysis

1. SUPPLEMENTARY METHODS

2. SUPPLEMENTARY FIGURES

Supplementary Figure S1.
Flowchart of the lyso-Gb3 screening and gene analysis in male patients

Supplementary Figure S2.
Flowchart of the lyso-Gb3 screening and gene analysis in female patients

3. SUPPLEMENTARY TABLES

Supplementary Table S1.
PCR primer sets

Supplementary Table S2.
RT-PCR protocol

Supplementary Table S3.
PCR protocol

Supplementary Table S4.
Clinical manifestations in women in the lyso-Gb3 positive group with normal α-Gal A and no *GLA* mutations, as detected by ordinary gene analysis

Supplementary Table S5.
GLA intronic GVUS in women in the lyso-Gb3 positive group with normal α-Gal A activity

Supplementary Table S6.
Clinical characteristics of patients in the lyso-Gb3 negative group with low α-Gal A activity who underwent ordinary gene analysis

Supplementary Table S7.
Intronic *GLA* variants in patients with GVUS in the lyso-Gb3 negative group with low α-Gal A activity and the c.−10C>T mutation

4. SUPPLEMENTARY ACKNOWLEDGMENTS

SUPPLEMENTARY METHODS

Measurement of plasma lyso-Gb3

Analyses were conducted at GlycoPharma (Oita, Japan). Lyso-Gb3 (Sigma-Aldrich, St. Louis, MO, USA) was further purified by HPLC to a maximum of purity and weighed for use as a standard. A glycine derivative of lyso-Gb3 (Gly-lyso-Gb3) was synthesized and purified for use as an internal standard.[40] Aliquots (40 µl) of plasma samples and 10 µl of 40 ng/ml Gly-lyso-Gb3 were mixed with 800 µl chloroform/methanol (2:1 [v/v]), and lipids were extracted by adding 200 µl of 1.5% formic acid and centrifuging at 6 500 × *g* using a Personal Centrifuge (Gyrogen, Seoul, Korea) for 5 min. Next, 200 µl of the clear upper layer were transferred to another tube and dried in a vacuum centrifuge. Samples were redissolved in water and extracted with water-saturated 1-butanol. The extracts were diluted with methanol and analyzed by UPLC-MS/MS. Quantitative UPLC-MS/MS analysis was performed on a Xevo TQD triple-quadrupole mass spectrometer equipped with an Acquity UPLC system (Waters, Milford, MA, USA). An Acquity HSS T3 column (1.8 µm, 2.1 × 50 mm; Waters) was equilibrated with 50% methanol containing 0.1% formic acid. A flow rate of 0.6 ml/min was selected, and elution was started with a linear gradient from 50 to 100% methanol in 0.8 min. Next, 100% methanol was used until 2.8 min, and the starting condition was applied. MS conditions were as follows: positive electrospray ionization, spray voltage, 3.5 kV; desolvation gas flow, 900 l/h; cone gas flow, 50 l/h; desolvation temperature,

300 °C; source temperature, 150 °C; cone voltage, 55 V. The following multiple-reaction monitoring transitions were detected: *m/z* 786.4 to 282.3 (lyso-Gb3) and *m/z* 843.4 to 339.3 (Gly-lyso-Gb3). The concentration of plasma lyso-Gb3 was calculated from a linear calibration curve generated by plotting the ratio of the peak area of lyso-Gb3 to that of Gly-lyso-Gb3 versus the actual concentration of the lyso-Gb3 standard in control plasma.

Gene analysis

DNA and RNA were extracted from white blood cells using a QIAamp DNA Blood Mini Kit (Qiagen, Tokyo, Japan) and PAXgene Blood RNA Kit, respectively. Human *GLA* consists of seven exons; exons 1 and 7 were amplified from genomic DNA by polymerase chain reaction (PCR), and a fragment containing the full-length exons 2–6 and all exon-intron boundaries was obtained by reverse transcription (RT) PCR using total RNA. PCR and RT-PCR were performed under the conditions shown in **Tables S1** and **S2**. Sequencing was performed as previously described.[14,18] When ordinary gene analysis revealed no mutations in females with a positive lyso-Gb3 screening result, we searched for long deletions using multiplex ligation-dependent probe amplification (MLPA) analysis and by assessing intronic mutations. Patients with negative lyso-Gb3 screening results and low α-Gal A activity were assessed for intronic mutations. MLPA was carried out following the manufacturer’s instructions using SALSA MLPA P159-A5 GLA Probemix (MRC-Holland, Amsterdam, the

Netherlands). PCR products were run on an ABI 3130xl Genetic Analyzer using GeneScan ROX 500 size standards (Applied Biosystems, Waltham, MA, USA). The data were normalized and analyzed using the Coffalyser NET software (MRC-Holland). PCR for introns was performed under the conditions shown in **Tables S1** and **S3**.

REFERENCE

40. Krüger R, Tholey A, Jakoby T, et al. Quantification of the Fabry marker lysoGb3 in human plasma by tandem mass spectrometry. *J Chromatogr B Analyt Technol Biomed Life Sci* 2012;883–884:128–135.

SUPPLEMENTARY FIGURES

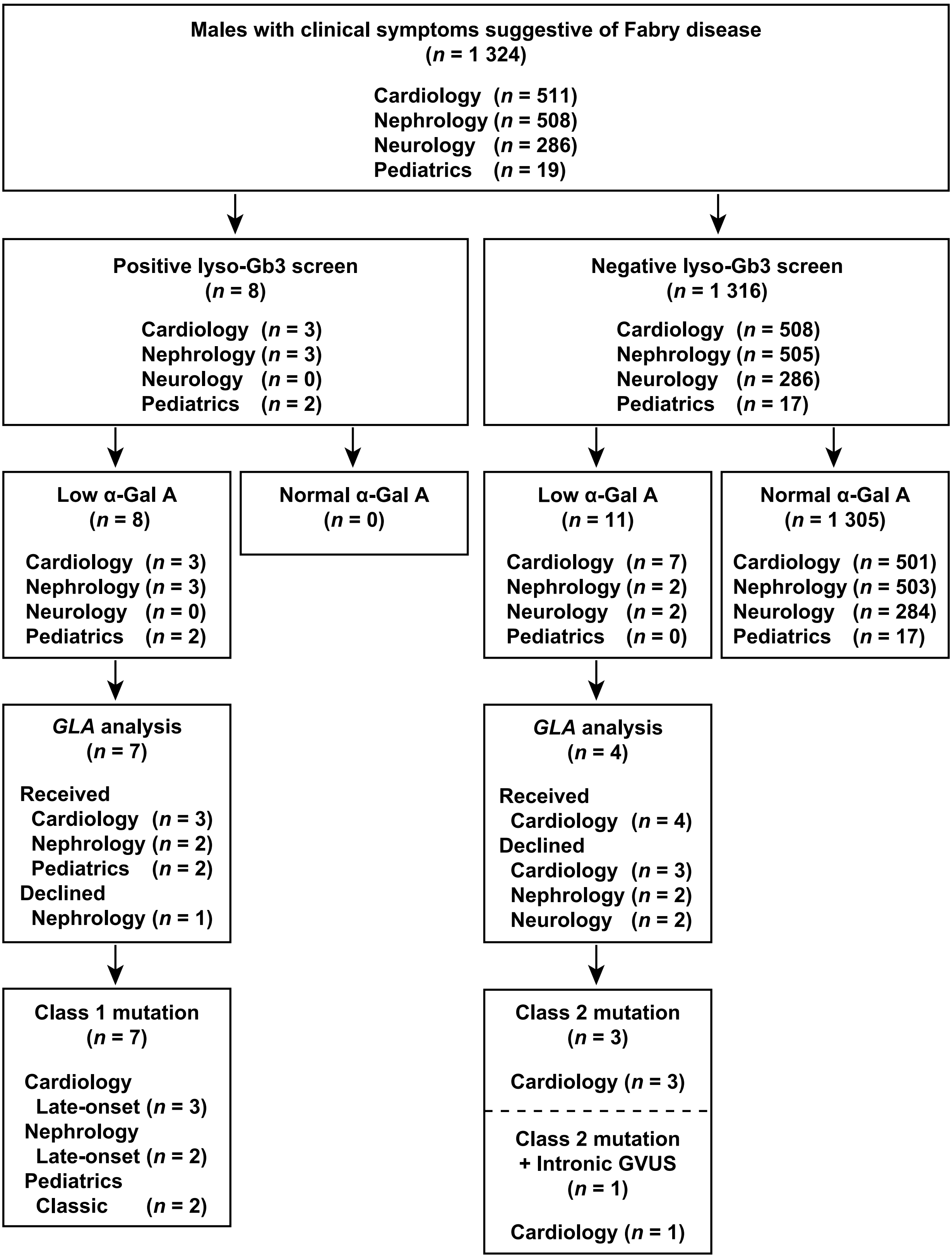

Supplementary Figure S1. Flowchart of the lyso-Gb3 screening and gene analysis in male patients

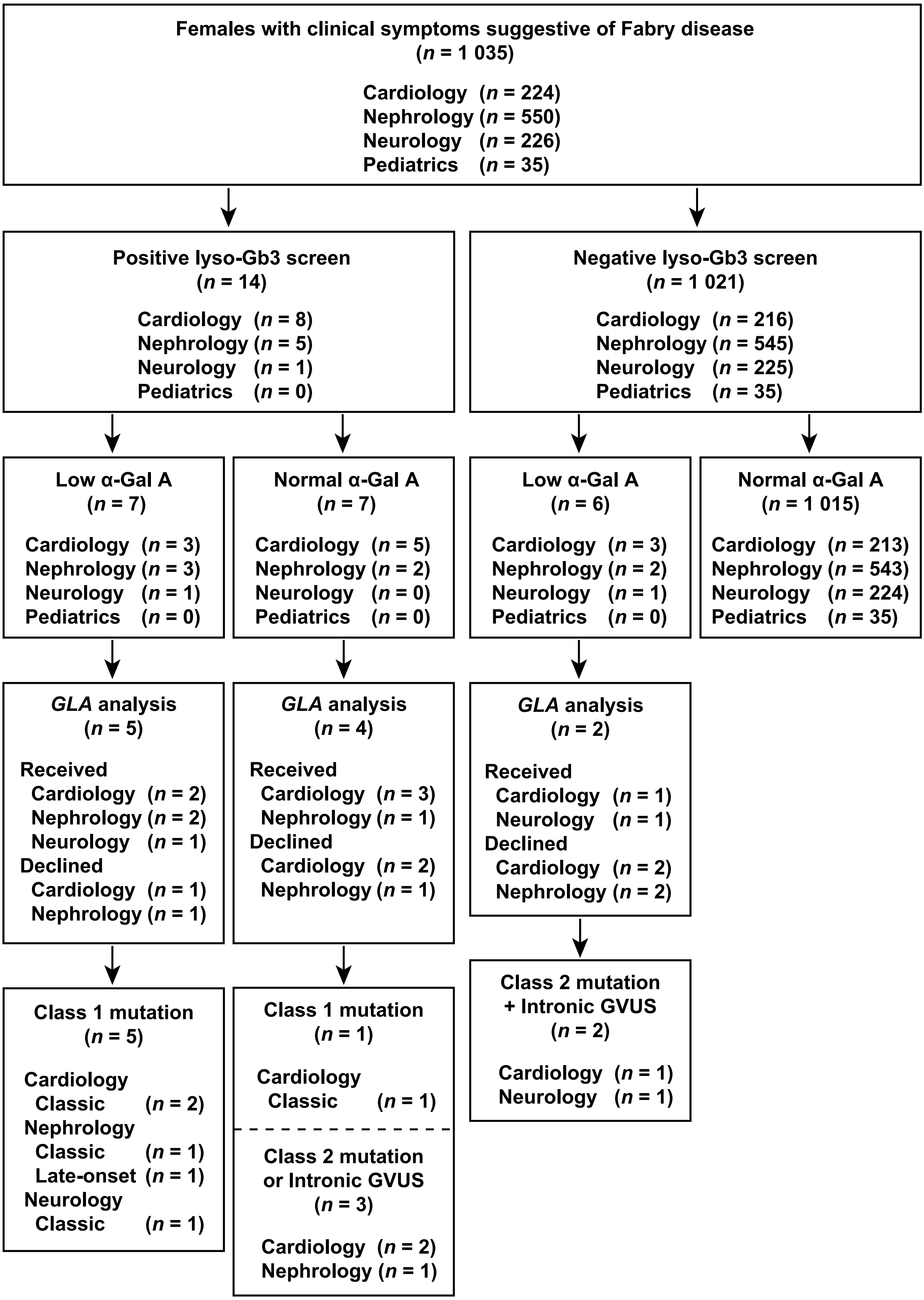

Supplementary Figure S2. Flowchart of the lyso-Gb3 screening and gene analysis in female patients

SUPPLEMENTARY TABLES

Supplementary Table S1. PCR primer sets

Name	Region	Sense	Antisense
cDNA*	c.147–1169	5′-CTTCATGTGCAACCTTGACTGCCAG-3′	5′-ACAGGGAGGAGCTGTGTGATGAAGC-3′
Ex1*	g.1057–1447	5′-CCAGTTGCCAGAGAAACAA-3′	5′-GAGACTCTCCAGTTCCC-3′
IVS1.a†	g.1398–2222	5′-CCCTTTGCTTTTCCATGTGT-3′	5′-TTGCTCTGTAAGCGCAGTTG-3′
IVS1.b†	g.1969–2777	5′-GGAAGGTTGACATGGAGGAA-3′	5′-TTGCCATTAGCCAAACATGA-3′
IVS1.c†	g.2642–3600	5′-CCAGAAATAATCATGGTTTAAATGTT-3′	5′-GTATTATCTTCAGGTAGGAAAGGACTT-3′
IVS1.d†	g.3477–4319	5′-AATACAGTGGTTCTTCCTATGTGTTGG-3′	5′-GGTTTTGCATTTTGGCTAGGCTA-3′
IVS1.e†	g.4173–5041	5′-TTTGTAATGTTGTGCCAAGTTCC-3′	5′-ACCTCCCATTTATTAGGCACCTT-3′
Exon2†	g.4902–5475	5′-GGAATATTAACGGGATAAGAGAGA-3′	5′-ACTTTTAATAGAGTTGGGGTTTCA-3′
IVS2.a†	g.5321–6189	5′-TACAGAAGCTTGTTTAGAAACAGC-3′	5′-AAGGTTATATGGAAATACATGCAC-3′
IVS2.b†	g.6066–6699	5′-ATGTTTTGTAATAGCTCTTGAGGC-3′	5′-AATCTGCTCATTGGCTATAAATCT-3′
IVS2.c†	g.6529–7295	5′-CTTGTAATCCGCCCACCTT-3′	5′-CCCTAGCTTCAGTCCTTTGCT-3′
Ex3–4	g.7075–8510	5′-TCAGCAGAACTGGGGGATT-3′	5′-AGTAACGTTGGACTTTGAAGG-3′
Exon4†	g.8119–8612	5′-GGAAGCTGAGACAGAAGAGT-3′	5′-AGACACAAGGATGACTTTCC-3′
IVS4.a†	g.8530–9355	5′-CCAGGTTCCAACCACTTCTC-3′	5′-CCCTGCCCTCATGAAACTTA-3′
IVS4.b†	g.9224–10138	5′-AGCCCTCTGTCCATTCATTCT-3′	5′-TAATTGGGCTGTGAAAACAGA-3′
Ex5–7	g.10075–11301	5′-CATCTCACAAGGATGTTAGT-3′	5′-CAGGAAGTAGTAGTTGGCAAT-3′
Ex7*	g.10881–11301	5′-ACAAGTGCTTGATAGTTCTGA-3′	5′-CAGGAAGTAGTAGTTGGCAA-3′

Primer sets for ordinary gene analysis were previously described.[20] Entire introns were covered by the following primer sets; Ex1 for 2% of intron 1 from IVS1+1, †primer sets[35] for the remaining region of intron 1, and for introns 2 and 4, Ex3–4 for intron 3, Ex5–7 for introns 5 and 6.

Supplementary Table S2. RT-PCR protocol

**RT**	
ReverTra Ace -α- (μl)	1
5× RT buffer (μl)	4
dNTP mix (10 mM) (μl)	2
RNase inhibitor (μl)	1
Random primer (25 pmol/μl) (μl)	1
RNA (240 ng) (μl)	x
RNase-free water (μl)	11 – x
Final volume (μl)	20
Annealing	30 °C, 10 min
Extension	42 °C, 20 min
Inactivation	99 °C, 5 min
Final hold	4 °C
**PCR**	
RT reaction sample (μl)	4.2
KOD -Plus- Neo (μl)	1
10× PCR buffer (μl)	5
$MgSO_4$ (25 mM) (μl)	3
dNTP mix (2 mM) (μl)	5
Sense primer (100 pmol/μl) (μl)	0.5
Antisense primer (100 pmol/μl) (μl)	0.5
Sterilized water (μl)	30.8
Final volume (μl)	50
Pre-denaturation	94 °C, 2 min
Denaturation	98 °C, 10 s
Annealing	68 °C, 20 s
Extension	68 °C, 20 s
Number of cycles	36
Final hold	4 °C

ReverTra Ace -α- (FSK-101; Toyobo, Osaka, Japan)

KOD -Plus- Neo DNA polymerase (KOD-401; Toyobo)

Thermal Cycler Dice Touch (Takara Bio, Shiga, Japan)

Supplementary Table S3. PCR protocol

	**Ex1**	**IVS1.a**	**IVS1.b**	**IVS1.c**	**IVS1.d**
**PCR mixture**					
KOD -Plus- Ver.2 (µl)	1	1	1	1	-
10× PCR buffer (µl)	5	5	5	5	-
$MgSO_4$ (25 mM) (µl)	2	2	2	3	-
10× PCR enhancer (µl)	-	5	5	-	-
LA *Taq* (µl)	-	-	-	-	0.5
10× LA buffer (µl)	-	-	-	-	5
$MgCl_2$ (25 mM) (µl)	-	-	-	-	5
dNTP mix (2 mM) (µl)	5	5	5	5	8
Sense primer (50 pmol/µl) (µl)	1	1	1	1	1
Antisense primer (50 pmol/µl) (µl)	1	1	1	1	1
DNA (100 ng) (µl)	x	x	x	x	x
Sterilized water (µl)	35 − x	30 − x	30 − x	34 − x	29.5 − x
Final volume (µl)	50	50	50	50	50
**PCR temperatures and cycles**					
Pre-denaturation	94 °C 2 min	94 °C 2 min	94 °C 2 min	94 °C 2 min	94 °C 2 min
Denaturation	96 °C 10 s	94 °C 30 s	94 °C 30 s	98 °C 10 s	94 °C 10 s
Annealing	55 °C 30 s	57 °C 30 s	57 °C 30 s	56 °C 30 s	68 °C 30 s
Extension	68 °C 30 s	68 °C 1 min	68 °C 1 min	68 °C 1 min 10 s	68 °C 30 s
Number of cycles	34	33	33	35	34
Final hold	4 °C	4 °C	4 °C	4 °C	4 °C

Supplementary Table S3, continued. PCR protocol

	IVS1.e	Exon2	IVS2.a*		IVS2.b
**PCR mixture**					
KOD -Plus- Ver.2 (µl)	1	1	1.5		0.5
10× PCR buffer (µl)	5	5	5		5
$MgSO_4$ (25 mM) (µl)	2	3	2		3
10× PCR enhancer (µl)	5	-	-		-
LA *Taq* (µl)	-	-	-		-
10× LA buffer (µl)	-	-	-		-
$MgCl_2$ (25 mM) (µl)	-	-	-		-
dNTP mix (2 mM) (µl)	5	5	5		5
Sense primer (50 pmol/µl) (µl)	1	1	1		1
Antisense primer (50 pmol/µl) (µl)	1	1	1		1
DNA (100 ng) (µl)	x	x	x		x
Sterilized water (µl)	30 − x	34 − x	34.5 − x		34.5 − x
Final volume (µl)	50	50	50		50
**PCR temperatures and cycles**					
Pre-denaturation	94 °C<br>2 min	94 °C<br>2 min	94 °C<br>2 min	-	96 °C<br>2 min
Denaturation	94 °C<br>30 s	94 °C<br>10 s	94 °C<br>15 s	94 °C<br>15 s	96 °C<br>30 s
Annealing	58 °C<br>30 s	56 °C<br>30 s	59–53 °C<br>15 s	55 °C<br>15 s	57 °C<br>30 s
Extension	68 °C<br>1 min	68 °C<br>30 s	68 °C<br>45 s	68 °C<br>3 min 45 s	68 °C<br>45 s
Number of cycles	33	31	7	25	30
Final hold	4 °C	4 °C	-	4 °C	4 °C

*Touchdown PCR consisted of 7 cycles using the thermocycling conditions shown in column 3, followed by 25 cycles using the conditions shown in column 4.

Supplementary Table S3, continued. PCR protocol

	IVS2.c	Ex3–4	Exon4	IVS4.a	IVS4.b
**PCR mixture**					
KOD -Plus- Ver.2 (µl)	-	1.5	1	-	1
10× PCR buffer (µl)	-	5	5	-	5
$MgSO_4$ (25 mM) (µl)	-	3	2	-	2
10× PCR enhancer (µl)	-	-	-	-	-
LA *Taq* (µl)	0.5	-	-	0.5	-
10× LA buffer (µl)	5	-	-	5	-
$MgCl_2$ (25 mM) (µl)	5	-	-	5	-
dNTP mix (2 mM) (µl)	8	5	5	8	5
Sense primer (50 pmol/µl) (µl)	1	1	1	1	1
Antisense primer (50 pmol/µl) (µl)	1	1	1	1	1
DNA (100 ng) (µl)	x	x	x	x	x
Sterilized water (µl)	29.5 − x	33.5 − x	35 − x	29.5 − x	35 − x
Final volume (µl)	50	50	50	50	50
**PCR temperatures and cycles**					
Pre-denaturation	94 °C<br>2 min	94 °C<br>2 min	94 °C<br>2 min	94 °C<br>2 min	94 °C<br>2 min
Denaturation	94 °C<br>10 s	94 °C<br>15 s	94 °C<br>10 s	94 °C<br>10 s	98 °C<br>10 s
Annealing	68 °C<br>30 s	57 °C<br>30 s	55 °C<br>30 s	68 °C<br>30 s	57 °C<br>30 s
Extension	68 °C<br>30 s	68 °C<br>1 min 30 s	68 °C<br>30 s	68 °C<br>30 s	68 °C<br>1 min
Number of cycles	30	33	30	34	33
Final hold	4 °C	4 °C	4 °C	4 °C	4 °C

Supplementary Table S3, continued. PCR protocol

	Ex5−7	Ex7
**PCR mixture**		
KOD -Plus- Ver.2 (μl)	1.5	1
10× PCR buffer (μl)	5	5
$MgSO_4$ (25 mM) (μl)	3	2
10× PCR enhancer (μl)	-	-
LA *Taq* (μl)	-	-
10× LA buffer (μl)	-	-
$MgCl_2$ (25 mM) (μl)	-	-
dNTP mix (2 mM) (μl)	5	5
Sense primer (50 pmol/μl) (μl)	1	1
Antisense primer (50 pmol/μl) (μl)	1	1
DNA (100 ng) (μl)	x	x
Sterilized water (μl)	33.5 − x	35 − x
Final volume (μl)	50	50
**PCR temperatures and cycles**		
Pre-denaturation	94 °C<br>2 min	94 °C<br>2 min
Denaturation	94 °C<br>10 s	96 °C<br>10 s
Annealing	56 °C<br>30 s	55 °C<br>30 s
Extension	68 °C<br>30 s	68 °C<br>30 s
Number of cycles	33	34
Final hold	4 °C	4 °C

KOD -Plus- Ver.2 DNA polymerase (KOD-211; Toyobo)

LA *Taq* polymerase (RR002A; Takara Bio)

PCR_X Enhancer Solution (11495017; Invitrogen, Carlsbad, CA, USA)

Thermal Cycler Dice Touch (Takara Bio)

Supplementary Table S4. Clinical manifestations in women in the lyso-Gb3 positive group with normal α-Gal A and no *GLA* mutations, as detected by ordinary gene analysis

Clinical department	Patient no.	Lyso-Gb3 levels (ng/ml)	α-Gal A activity (nmol/h/ml)	Sex	Age (years)	Classic manifestation	Heart	Kidney	Central nervous system
Cardiology	14	18.5	10.9	Female	67	Hypohidrosis	LVH<br>Arrhythmia<br>Lamellar body	None	None
	15	8.4	8.5	Female	59	None	LVH<br>Arrhythmia<br>Lamellar body	G5A3<br>Lamellar body	None
Nephrology	16	7.8	4.7	Female	69	Hypohidrosis	Arrhythmia	G3aA3<br>Lamellar body	None

LVH: Left ventricular hypertrophy

Glomerular filtration rate category (ml/min/1.73 m^2): G3a, 45–59; G5, < 15[15]

Proteinuria category (g/gCr): A3, ≥ 0.50

Supplementary Table S5. *GLA* intronic GVUS in women in the lyso-Gb3 positive group with normal α-Gal A activity

				Patient no.		
	**Variants**	**Genome**	**dbSNP**	**14**	**15**	**16**
Intron 1	c.194+1562_+1563insC	g.2935_2936	-	+	+	+
	c.195−1854G>A	g.3240	-	+/−	+/−	−
	c.195−1424_−1423insT	g.3670_3671	-	+	+	+
	c.195−865_−864insC	g.4229_4230	-	+	+	+
	c.195−655_−654insC	g.4439_4440	-	+	+	+
	c.195−522G>A	g.4572	rs111386229	−	+/−	−
Intron 2	c.369+103_+104insC	g.5371_5372	-	+	+	+
	c.369+812C>T	g.6080	-	−	−	−
	c.369+990C>A	g.6257	rs1023431	−	+/−	−
	c.370−81_−77delCAGCC	g.7188_7192	rs5903184	−	+/−	−
Intron 3	c.547+419C>T	g.7865	rs146298823	+/−	+/−	−
Intron 4	c.639+393C>T	g.8805	rs782312990	−	−	−
	c.640−730A>G	g.9401	-	+	+	+
	c.640−302_−301insC	g.9829_9830	-	+	+	+
	c.640−255_−254CG>GC	g.9876_9877	-	+	+	+
	c.640−16A>G	g.10115	rs2071397	−	+/−	−
Intron 6	c.1000−22C>T	g.10956	rs2071228	−	+/−	−

Supplementary Table S6. Clinical characteristics of patients in the lyso-Gb3 negative group with low α-Gal A activity who underwent ordinary gene analysis

Clinical department	Patient no.	Lyso-Gb3 levels (ng/ml)	α-Gal A activity (nmol/h/ml)	Sex	Age (years)	Classic manifestation	Heart	Kidney	Central nervous system
Cardiology	17	0.5	3.4	Male	62	None	LVH<br>Arrhythmia	None	Stroke
	18	0.5	3.9	Female	75	None	LVH<br>Arrhythmia	G3bA2	White matter lesion
Neurology	19	0.5	3.9	Female	52	Acroparesthesia<br>Hypohidrosis	None	None	None

LVH: Left ventricular hypertrophy

Glomerular filtration rate category (ml/min/1.73 m^2): G3b, 30–44[15]

Proteinuria category (g/gCr): A2, 0.15–0.49

Supplementary Table S7. Intronic *GLA* variants in patients with GVUS in the lyso-Gb3 negative group with low α-Gal A activity and the c.−10C>T mutation

				Patient no.		
	**Variants**	**Genome**	**dbSNP**	**17**	**18**	**19**
Intron 1	c.194+1562_+1563insC	g.2935_2936	-	+	+	+
	c.195−1854G>A	g.3240	-	−	−	−
	c.195−1424_−1423insT	g.3670_3671	-	+	+	+
	c.195−865_−864insC	g.4229_4230	-	+	+	+
	c.195−655_−654insC	g.4439_4440	-	+	+	+
	c.195−522G>A	g.4572	rs111386229	+	+/−	+
Intron 2	c.369+103_+104insC	g.5371_5372	-	+	+	+
	c.369+812C>T	g.6080	-	−	+	−
	c.369+990C>A	g.6257	rs1023431	+	+/−	+
	c.370−81_−77delCAGCC	g.7188_7192	rs5903184	+	+/−	+
Intron 3	c.547+419C>T	g.7865	rs146298823	−	−	−
Intron 4	c.639+393C>T	g.8805	rs782312990	−	+/−	+/−
	c.640−730A>G	g.9401	-	+	+	+
	c.640−302_−301insC	g.9829_9830	-	+	+	+
	c.640−255_−254CG>GC	g.9876_9877	-	+	+	+
	c.640−16A>G	g.10115	rs2071397	+	+/−	+
Intron 6	c.1000−22C>T	g.10956	rs2071228	+	+/−	+

SUPPLEMENTARY ACKNOWLEDGMENTS

The following physicians and centers participated in this study: Hiroshi Kawaguchi, Department of Nephrology, Joban Hospital, Tokiwa Kai; Masatugu Sato, Kojinkai Kimachi Hospital; Kanemitsu Yamada, Oyokyo Kidney Research Institute; Akihiro Fujii, Department of Neurology, Saiseikai Shigaken Hospital; Atsushi Misao, Misao Surgical Hospital; Takahiro Maruta, Neurological Center, Kanazawa-Nishi Hospital; Yu Kano, Department of Neurology, The Jikei University School of Medicine; Manabu Iwata, Hirosaki Stroke and Rehabilitation Center; Takayuki Hidaka, Department of Cardiovascular Medicine, Hiroshima University Hospital; Hisashi Yonezawa, Department of Neurology and Gerontology, Iwate Medical University; Eiji Yamashita, Department of Cardiology, Gunma Prefectural Cardiovascular Center; Takahiro Tanaka, Showa General Hospital; Yoshiharu Taguchi, Department of Neurology, Toyama University Hospital; Masaki Matsunaga, Iwata City Hospital; Hitoshi Watanabe, Gifu Central Hospital; Sanami Kawada, Okayama Kyokuto Hospital; Takashi Shimozato, Nagoya Tokushukai General Hospital; Toru Kawai, Chuou Naika Clinic; Keiji Yamaguchi, Department of Neurology, Ichinomiya Nishi Hospital; Yasuhiro Morita, Ogaki Municipal Hospital; Hiroyuki Kinuno, Toyama Rosai Hospital; Masayoshi Ajioka, Department of Cardiovascular Internal Medicine, Tosei General Hospital; Tetsuro Takeda, Dokkyo Medical University Koshigaya Hospital; Tsuguhisa Hatano, Hatano Clinic; Tatsuji Kono, Soseikai General Hospital; Satoshi Sakai, Department of Cardiology, Faculty of Medicine, University of

Tsukuba; Mamoru Nobuhara, Department of Cardiology, Municipal Kosai Hospital; Yasushi Tanaka, Department of Cardiovascular Medicine, Yodogawa Christian Hospital; Eriko Kinugasa, Showa University, Northern Yokohama Hospital; Koji Ishiguro, Department of Neurology, National Hospital Organization, Takasaki General Medical Center; Yumiko Nakashima, Department of Pediatrics, Nagasaki University Hospital; Hiromitsu Tominaga, Kikugawa City General Hospital; Azumi Kumazawa, Omaezaki Municipal Hospital; Yukio Hiroi, National Center for Global Health and Medicine; Takashi Tokunaga, Nishinomiya Kyoritsu Neurological Hospital; Nobuyuki Murakami, Department of Pediatrics, Dokkyo Medical University Koshigaya Hospital; Hiroki Uozumi, Japanese Red Cross Medical Center; Koichi Node, Division of Cardiovascular, Saga University; Kazuhiro Aoki, Tokyo National Hospital; Toshiyuki Sugawara, Department of Cardiology, Aomori City Hospital; Shigemi Nagayama, Department of Neurology, Kanazawa Medical University; Akiko Kumagai, Department of Internal Medicine, Division of Cardiology and Memorial Heart Center, Iwate Medical University; Mikio Enomoto, Department of Laboratory, Toyooka Hospital; Kouji Kajinami and Minoru Wakasa, Department of Cardiology, Kanazawa Medical University; Toru Kubo, Department of Cardiology and Geriatrics, Kochi Medical School, Kochi University; Hitoshi Koito, Misugikai Otokoyama Hospital; Daisuke Fukumoto, Department of Cardiovascular and Respiratory Medicine, Shiga University of Medical Science; Kentaro Kohagura, Dialysis Unit University Hospital of the Ryukyus; Takako Iino, Akita University

Graduate School of Medicine; Toshiya Kataoka, Hyogo Prefectural Kakogawa Medical Center; Chisato Izumi, Department of Cardiology, Tenri Hospital; Masahiko Nakamura, Yamanashi Prefectural Central Hospital; Hideki Fujii, Division of Nephrology and Kidney Center, Kobe University Graduate School of Medicine; Fumihiro Tomoda, Faculty of Health Science, Fukui Health Science University; Yoichi Iwafuchi, Koseiren Sanjo General Hospital; Norie Tanaka-Saito, Department of Cardiology, Kushiro Kojinkai Memorial Hospital; Ohsuke Migita, St. Marianna University School of Medicine; Satoru Takeda, Hiraka General Hospital; Tadashi Kakio, Department of Cardiovascular Medicine, Kyoto Min-iren Chuo Hospital; Yuji Koide, Nagasaki University; Tomiyoshi Saito, Second Department of Internal Medicine, Shirakawa Kosei General Hospital; Gen Takezawa, Akiota Hospital; Koji Hontoku, Fukaya Red Cross Hospital; Nobuya Fujita, Nagaoka Red Cross Hospital; Seiji Okubo, Department of Neurological Science, Graduate School of Medicine, Nippon Medical School; Toshiichi Watanabe, Department of Neurology, Nakamura Memorial Hospital; Junya Kobayashi, Department of Vascular Neurology, National Hospital Organization Osaka Minami Medical Center; Toshiyuki Ohta, Department of Pediatric Nephrology, Hiroshima Prefectural Hospital; Toshinori Minato, Department of Pediatrics Director, Toyooka Hospital; Yusuke Kashiwagi, Hokkaido Cardiovascular Hospital; Shigeo Nakajima, Nakajima Cardiovascular Medicine Mental Clinic; Atsushi Izawa, School of Health Sciences, Shinshu University; Hiroyuki Yabata, Kohka Public Hospital; Shinichiro Nakamura, Koshigaya Municipal Hospital; Kazuhide Ohta,

Department of Pediatrics, Kanazawa Medical Center; Shuichiro Umetsu, Saiseikai Yokohama-shi Tohbu Hospital; Kentaro Mukai, Department of Cardiology, Aichi Medical University; Orie Ichikawa, Department of Cardiology, National Hospital Organization, Kure Medical Center; Mikiko Ohno, Department of Pharmacology, Shiga University of Medical Science; Yoshiki Ueno, Nagahama Red Cross Hospital; Ryo Yamamura, Obama Municipal Hospital; Shinji Hasegawa, Division of Cardiovascular, Japan Community Health Care Organization Osaka Hospital; Mitsuteru Koizumi, Department of Nephrology, Kyoto Medical Center, National Hospital Organization; Atsushi Wada, Kitasaito Hospital; Itaru Ebihara, Department of Nephrology, Mito Saiseikai General Hospital; Kumiko Muta, Nagasaki University Hospital; Keiichi Hirao, Shibagaki Dialysis Clinic Togoshi; Machiko Shinkai, Shinkai Family Clinic; Takaya Abe, Seitetsu Memorial Hospital; Tomoyuki Miyamoto, Dokkyo Medical University Koshigaya Hospital; Hiroo Kasahara, Department of Neurology, Gunma University Graduate School of Medicine; Akihiko Ogata, Department of Neurology, Hokkaido Neurosurgical Memorial Hospital; Yasuhiro Kumai, Departments of Cerebrovascular Disease and Neurology, Hakujuji Hospital, Fukuoka; Ryota Tanaka, Department of Neurology, Juntendo University; Kazuma Tsuto, Department of Neurology & Stroke Treatment, Kyoto First Red Cross Hospital; Akio Yokochi, Division of Nephrology, Kanto Rosai Hospital; Tetsuya Masaoka, Department of Neurosurgery, Mitoyo General Hospital; Nobuyuki Takahashi, Nara City Hospital; Yasuhiro Hasegawa, Department of Neurology, St. Marianna University School of Medicine; Yasutaka

Tajima, Department of Neurology, Sapporo City General Hospital; Shin Hisahara, Department of Neurology, Sapporo Medical University; Naoto Fujita, Department of Pediatrics, Hiroshima Red Cross Hospital & Atomic-bomb Survivors Hospital; Taku Nakagawa, Department of Pediatrics, Japanese Red Cross Society Himeji Hospital; Akihiko Maeda, Kochi Prefectural Hata-Kenmin Hospital; Shin Hosino, Kasugai Municipal Hospital; Yasutsugu Chinen, Department of Pediatrics, Faculty of Medicine, University of the Ryukyus; Yasufumi Ohtsuka, Department of Pediatrics, Faculty of Medicine, Saga University; Masaki Nii, Department of Cardiology, Shizuoka Children's Hospital; Takeshi Matsushige, Department of Pediatrics, Yamaguchi University Graduate School of Medicine; and Tomoaki Sano, Department of Pediatrics, Yamanashi Red Cross Hospital.
